# Left ventricular remodelling and prosthetic valve function after transcatheter aortic valve implantation: A serial cardiac magnetic resonance imaging study

**DOI:** 10.1186/1532-429X-17-S1-O85

**Published:** 2015-02-03

**Authors:** Constanze Merten, Hans Beurich, Dirk Zachow, Mohamed El-Mawardy, Gert Richardt, Mohamed Abdel-Wahab

**Affiliations:** 1 Cardiology, Herzzentrum Bad Segeberg, Bad Segeberg, Germany; 2 Radiology, Segeberger Kliniken, Bad Segeberg, Germany

## Background

Transcatheter aortic valve implantation (TAVI) has become a common procedure for high-risk patients with severe aortic stenosis. Aortic regurgitation (AR) is commonly seen after TAVI, but little is known about how it evolves over time. Similarly, the impact of TAVI on left ventricular (LV) function, LV volumes and mass is not well defined.Paravalvular aortic regurgitation (AR) is frequent after TAVI. Yet, little data is available about temporal changes of AR and the impact of TAVI on left ventricular (LV) function and dimensions.

We used cardiac MRI to evaluate LV function, volumes and mass, the occurrence and degree of AR in the early and medium-term follow-up after TAVI.

## Methods

In 81 patients who underwent transfemoral TAVI using the Medtronic Corevalve prothesis or the Edwards Sapien valve we performed baseline cardiac MRI at a median of 11 days after TAVI and follow-up MRI 6 months later.

LV volumes and function were assessed using standard cine MRI sequences. Additionally, phase contrast imaging was conducted to measure aortic regurgitation.

A calculated RF of 1%-15% was graded I (mild), 16%-30% was graded II (moderate), 31%-50% was graded III (moderate to severe) and > 50% was graded IV (severe), a RF <1% was classified as no AR.

## Results

The median age of the evaluated patients was 81 years (range 61 - 90y) and 60% were women. At baseline MRI, the median LV ejection fraction was 58.0% (range 22.1-71.7%), which improved significantly at follow-up to 63.4% (range 24.0 - 73.7%, p<0.0001).

In addition, a significant reduction of LV end-diastolic volume (139.7 ml , range 69.4-260.7ml, vs. 129.7ml, range 78.7-272.2ml, p=0.0017) and of LV mass (151.4 ± 34.1 g vs. 139. ± 33.5 g, p<0.0001) was observed.

Baseline MRI identified no AR in 15 patients, grade I AR in 55, grade II in 9 and grade III AR in 2 patients. The median aortic RF was 4.2% (range 0.1 to 39.0%) at baseline and remained stable with 5.0% (range 0.0 to 41.9%, p=0.02) at follow-up.

The changes in LV function, volume and mass observed in the oberall cohort were not present in the subgroup of patients with relevant AR (grade II and III, n=11) at baseline MRI: the mean ejection fraction only showed a trend to improvement (48.8 ± 15.7% vs. 50.9 ± 15.0%, p=0.08) whereas the mean LV end-diastolic volume (183.3 ± 50.1 ml vs. 184.6 ± 55.9 ml, p=0.90) and LV mass (174.9 ± 25.8 g vs. 172.8 ± 41.3 g, p=0.84) were almost identical at baseline and follow-up.

## Conclusions

Using cardiac MRI in TAVI patients, a significant improvement of left ventricular function, volume and mass can be documented after TAVI. Aortic regurgitation which is mostly mild is commonly seen in patients treated with TAVI. The aortic regurgitant fraction remained stable over time. Yet, positive left ventricular remodelling could only be observed in patients with no or mild AR after TAVI whereas no changes occured in the subgroup of patients with relevant AR.

## Funding

None.

